# Retraction: IKBKE Phosphorylation and Inhibition of FOXO3a: A Mechanism of IKBKE Oncogenic Function

**DOI:** 10.1371/journal.pone.0289330

**Published:** 2023-07-25

**Authors:** The PLOS ONE Editors

The Moffitt Cancer Center investigated this article [1] and determined that there was evidence of data fabrication and/or falsification in the following figures:

- Fig 1B, HA-FOXO3a panel
- Fig 1C, ChIP assay panel
- Fig 3E, pFOXO3A-S644panel, column 1
- Fig 5A, Myr-IKBKE panel
- Fig 5C, IKBKE panel, lane 2
- Fig 5G, HA-FOXO3a panel

Following their findings of misconduct the Moffitt Cancer Centre recommended retraction of [1].

The PLOS Editors also noted image irregularities in the additional panels listed below. PLOS did not follow up on these additional concerns.

- Fig 1D, Myc-IKBKE panel
- Fig 2A, HA-FOXO3a panel and Myr-IKBKE panel
- Fig 2B, left HA-FOXO3a panel and right Myr-IKBKE panel
- Fig S3 IKKα/β IKBKE panel

The authors did not respond to editorial communications regarding the above concerns and did not provide any underlying data.

In light of the outcome of the institutional investigation and the concerns affecting multiple figure panels that question the integrity of these data, the *PLOS ONE* Editors retract this article.

WT responded but expressed neither agreement nor disagreement with the editorial decision. JPG, SS, YX, CS, and JQC either did not respond directly or could not be reached.
